# Emotional and Behavioral Impact of Biological Parents Contact with their Foster Care Children: A cross-sectional study of Mental Health Outcomes

**DOI:** 10.1192/j.eurpsy.2026.11116

**Published:** 2026-07-31

**Authors:** I. Farmakopoulou, L. Kyriakou, M. Theodoratou

**Affiliations:** 1 Sciences of Education and Social Work, University of Patras; 2 Social Sciences, Hellenic Open University, Patras, Greece; 3 Health Sciences, Neapolis university Pafos, Pafos, Cyprus

## Abstract

**Introduction:**

Foster care in Greece provides temporary protection and stability for children separated from their biological families. Since **Law 4538/2018**, maintaining parental contact has become a legal and therapeutic priority, yet its psychological effects remain insufficiently explored.

**Objectives:**

To investigate the **frequency, nature, and psychological consequences** of contact between foster children and their biological parents, and to evaluate its influence on the children’s **emotional well-being and behavior**.

**Methods:**

A **cross-sectional quantitative study** was conducted with **30 foster families** and **13 social workers** from Child Protection Units in Attica. Data were collected using the **Strengths and Difficulties Questionnaire (SDQ)** and a **self-designed questionnaire**. Analysis employed **IBM SPSS 24**, descriptive statistics, and the **Mann–Whitney U test**.

**Results:**

Children were mainly **3–8 years old (70%)**, with **monthly supervised face-to-face contact (60%)**, usually with mothers (58%). Following visits, **63%** of foster parents reported **anxiety**, **57%** mood changes, and **48%** sleep or regressive behaviors. Although **56.7%** rated the child’s overall emotional health as good, social workers tended to report lower adjustment scores.

**Conclusions:**

**Supervised and psychologically supported contact** is essential to foster **stability and resilience** in children under Greek foster care.

**Disclosure of Interest:**

None Declared

